# Predicting Internet risks: a longitudinal panel study of gratifications-sought, Internet addiction symptoms, and social media use among children and adolescents

**DOI:** 10.1080/21642850.2014.902316

**Published:** 2014-03-31

**Authors:** Louis Leung

**Affiliations:** ^a^ Centre for Communication & Public Opinion Survey, School of Journalism & Communication, The Chinese University of Hong Kong, Shatin, NT, Hong Kong

**Keywords:** adolescents and children, gratifications-sought, Internet addiction symptoms, Internet risks, social media

## Abstract

This study used longitudinal panel survey data collected from 417 adolescents at 2 points in time 1 year apart. It examined relationships between Internet risks changes in Time 2 and social media gratifications-sought, Internet addiction symptoms, and social media use all measured at Time 1. By controlling for age, gender, education, and criterion variable scores in Internet addiction at Time 1, entertainment and instant messaging use at Time 1 significantly predicted increased Internet addiction measured at Time 2. The study also controlled for demographics and scores of criterion variables in Internet risks: targeted for harassment, privacy exposed, and pornographic or violent content consumed in Time 1. Gratifications-sought (including status-gaining, expressing opinions, and identity experimentation), Internet addiction symptoms (including withdrawal and negative life consequences), and social media use (in particular, blogs, and Facebook) significantly predicted Internet risk changes in Time 2. These findings suggest that, with their predictive power, these predictors at Time 1 could be used to identify those adolescents who are likely to develop Internet addiction symptoms and the likelihood of experiencing Internet risks based on their previous gratifications-sought, previous addiction symptoms, and their habits of social media use at Time 1.

## 1. Introduction

Social media is a group of Internet-based applications that build on the technological foundations of Web 2.0 which allows the creation and exchange of user-generated content. Social media takes on many different forms which involves both web-based and mobile technologies, including Internet forums, email, instant messaging (IM), social network services, blogs, and microblogs, to name but a few. Users can publish their own diaries on their personalized social network sites, post photos or videos, express opinions, meet other users, and establish communities based on shared interests (Livingstone & Brake, 2010).

Today, going beyond being popular among adolescents, social media has now evolved into a much-debated public concern because of excessive or maladaptive use. In fact, a growing collection of the literature demonstrates a consistently positive relationship between adolescents' Internet consumption and Internet risks (Leung & Lee, 2012a; Livingstone & Helsper, 2007). Being described as experiences online that had bothered users or made users feel uncomfortable or upset (Livingstone & Haddon, 2008), some of the Internet risks facing young people may include exposure to pornographic or violent material (Sabina, Wolak, & Finkelhor, 2008), being targeted for harassment (Kiriakidis & Kavoura, 2010), cyber-bullying (Erdur-Baker, 2010), sexual solicitation (Baumgartner, Valkenburg, & Peter, 2010), and Internet addiction (Young, 1998a).

But what drive adolescents' excessive use of social media? Past research in the gratifications or benefits of computer-mediated technologies such as the Internet (Papacharissi & Rubin, 2000), Facebook (Nadkarni & Hofmann, 2012; Yang & Brown, 2013), electronic bulletin boards (Dimmick, Kline, & Stafford, 2000), and user-generated content (Leung, 2009), among others, suggests broad motivations. These motivations include information exchange, conversation and socializing, entertainment, escape and diversion, reassurance, and fashion. With these motivations, adolescents often lose control in the amount of use which may lead to Internet addiction.

Internet addiction has been characterized as the use of the Internet to escape from negative feelings, continued use of the Internet despite the desire to stop, experience of unpleasant emotions when Internet use is impossible, thinking about the Internet constantly, and the experience of any other conflicts or self-conflicts due to Internet use (Young, 1998a, 1998b). This study adopts the uses and gratifications framework to establish the socio-psychological origins of needs in social media use among adolescents. This provides the basis to examine how these gratifications are related to different patterns of social media use, resulting in need gratifications and possible consequences such as Internet addiction and vulnerability to Internet risks. One may conceptualize the study with Internet addiction symptoms as the outcomes or the ultimate dependent variables with gratifications-sought, intensity of social media use, and Internet risks as predictors. Despite that Internet addiction is in effect a form of Internet risk, treating Internet addiction symptoms as predictors may help identify symptoms as early warning signs for teachers and parents to take precautionary actions before symptoms eroding into Internet risks.

However, one limitation of previous research in this area is the absence of longitudinal methods to help establish causal relationships and strengthen the findings of existing cross-sectional reports (Livingstone & Helsper, 2010). Accordingly, this study takes a longitudinal panel study approach to test whether gratifications-sought, Internet addiction symptoms, and social media use are related to Internet risks using two waves of data collected one year apart. Although experimental research is unparalleled for establishing causal relationships in the short term, a longitudinal framework is useful for identifying patterns that will help illuminate developmental trends in the roles of social media use and Internet addiction symptoms in adolescents' perceived Internet risks. This framework also allows us to improve the rigor of our statistical analyses by using controls such as gender, age, education, and Internet risks experienced at the outset of the study in Time 1 predicting Internet addiction and Internet risks in Time 2 at the conclusion of the study.

## 2. Literature review

### 2.1. Uses and gratifications

The uses and gratifications approach assumes that social and psychological motivations may cause people to turn to media for companionship and other gratifications. In this respect, the audience is often attempting to satisfy certain social and psychological needs such as surveillance, information-seeking, entertainment, personal identity, or companionship (Dimmick, Sikand, & Patterson, 1994; Rubin, 1983). The general theoretical conclusion of many uses and gratifications studies is that the behavior of media gratification seeking is regarded as active, goal-directed, and utility-driven (Blumler, 1979; Katz, Blumler, & Gurevitch, 1974). This utilitarian view of media use can be conceptually applicable to people's motives associated with the use of social media (such as IM, blogs, Facebook, and online games). One goal of this study is to explore a wide range of motivations connected to Internet use, especially with social media which users can identify as unique for adolescents (Quan-Haase & Young, 2010). Thus, the more users are gratified with these needs, the more they will use social media. When the level of social media use becomes excessive, it is logical to assume that the higher the probability they will be addicted and exhibit Internet addiction symptoms. As a consequence, users will more likely to suffer from Internet risks. In studying Internet risks, previous research has explored privacy and security risks for online shopping (Miyazaki & Fernandez, 2001), taking risky opportunities in youthful content creation (Livingstone, 2008a), and balancing opportunities and risks in teenagers’ use of the Internet (Livingstone & Helsper, 2010), almost no study has focused on the relationships between Internet risks and gratifications-sought of social media use and Internet addiction symptoms. With this rationale, this study examines how gratifications-sought in social media use can predict Internet addictions and subsequently Internet risks experienced one year later.

### 2.2. Internet addiction as a concept for study

Traditionally, the concept “addiction” was based on a medical model and is properly reserved for bodily and psychological dependence on a physical substance – and not a behavioral pattern. Recent research has argued that addiction should be widened to cover a broader range of behaviors (Byun et al., 2009; Griffiths, 1998). Griffiths (1996) proposed, as a subset of behavioral addiction, the concept of technological addiction, which is operationally defined as human–machine interaction and is non-chemical in nature.

Derived from criteria associated with pathological gambling in the *Diagnostic and Statistical Manual* – *Fourth Edition* (*DSM-4*) (American Psychiatric Association, 1994), Young (1998a) presented a definition for Internet-related disorders, called problematic Internet use. This classic definition requires that individuals meet five of eight symptoms for Internet addiction to qualify as an addict. These symptoms include: (1) preoccupation with the Internet, (2) need for longer periods of time online, (3) repeated attempts to reduce Internet use, (4) withdrawal symptoms when reducing Internet use, (5) time management issues, (6) environmental distress (family, school, work, and friends), (7) deception around time spent online, and (8) mood modification through Internet use (Young, 1998a).

Results from past research indicate that Internet use had interfered with participants' academic work (Iskender & Akin, 2010; Leung & Lee, 2012b) and professional performance or their social lives (Young, 1998b). Others described skipping sleep, ignoring family responsibilities, and showing up late for work to fulfill their desire to visit chat rooms and surf the web (Lam, Peng, Mai, & Jing, 2009). The evidence points to a psychological disorder (Hur, 2006), so researchers probed further and found that participants' habits met the criteria for impulse control disorder, a mental illness characterized by an uncontrollable desire to perform a behavior. Drawing on the insights from the definition and previous research, this study seeks to identify the types of Internet addiction symptoms among adolescents and explores how these symptoms measured at the outset relate to Internet risks reported one year later.

### 2.3. Internet risks

In this study, three commonly experienced Internet risks by children and adolescents are examined, namely Internet harassment, privacy exposed, and pornographic and violent content consumed. Internet harassment is an overt, intentional act of aggression toward another person online. Actions can take the form of purposefully harassing or embarrassing someone else or making rude or nasty comments toward someone else while online (Ybarra, Diener-West, & Leaf, 2007). Privacy exposed refers to the inability to control the information about oneself over the Internet especially regarding who can access that information. Increasingly, whether email can be read by third parties without consent or whether third parties can continue to track the websites someone has visited for marketing purpose has become a growing concern. Livingstone and Helsper (2007) found that making friends online has attracted particular attention as a risky behavior, especially when this leads to offline meetings, as has giving out personal or private information (such as email addresses, phone numbers, home addresses, and credit card information) online.

For decades, previous research on Internet risks indicates that parents and others have been consistently concerned about the potentially harmful influences of exposure to pornographic and violent content (Liau, Khoo, & Ang, 2005; Livingstone & Helsper, 2007; Peter & Valkenburg, 2006; Ybarra et al., 2007). A survey in Sweden (Carlsson, 2006) asked adults what they perceive to be the factors that lead to violence in their society. While alcohol and drugs were the highest (90%), both TV and the Internet were listed by 60% of respondents as having a strong and significant influence. Similarly, in a national poll in the USA, more than 85% considered the Internet to be more of a risk problem for their children than TV (Common Sense Media, 2006).

In light of the fact that different social media activities may bring different opportunities and risks (Livingstone, 2008a), this study limited its scope and examined the relationships between Internet risks and the use of four activities online that children and adolescents often spend most of their time on (i.e. the use of IM, blogs, Facebook, and online games). Taken as a whole, the literature to date raises the question of whether social media use can cause adolescents to develop addiction symptoms as well as erode these forms of addiction into health risks. To expand on this line of inquiry, this study examines the impact of gratifications-sought from social media use, Internet addiction symptoms experienced, and social media use on Internet risks one year apart. The assessed criterion variables were being targeted for harassment, having privacy exposed, and exposure to pornographic or violent content. Specifically, the study was conducted to examine whether the above factors measured in Time 1 predicted changes in adolescents' perceived Internet addiction symptoms and Internet risks exhibited in Time 2. Furthermore, it also raises the question of whether effects from social media use (namely IM, blogs, Facebook, and online games) may vary from one medium to another. In other words, the question we ask is: Among which respondents were the surges in Internet risks most pronounced? Thus, we proposed the following research questions:

RQ1: What are the significant surges or declines, if any, in the key measures among panel respondents over the one year period?

RQ2: To what extent did social media use (i.e., IM, blogs, Facebook, and online games) and gratifications-sought in social media use explain individual level changes in Internet addiction symptoms over the course of the following year?

RQ3: To what extent did social media use (i.e., IM, blogs, Facebook, and online games) and gratifications-sought in social media use, and Internet addiction symptoms explain individual level change in Internet risks (i.e., being targeted for harassment, having privacy exposed, and pornographic or violent content consumed) over the course of the following year?

## 3. Method

### 3.1. Sample and sampling procedure

To test the relationships over time between predictor variables (i.e. gratifications-sought, Internet addiction symptoms, and social media use) and the Internet risks criterion variables (i.e. being targeted for harassment, having privacy exposed, and pornographic or violent content consumed), survey data were collected at two points in time a year apart using a face-to-face structured questionnaire interview. At Time 1, during the period from December 2008 to February 2009, a random sample of eligible households was sent a postcard invitation from the author with a short description of the study. Respondents were eligible members of randomly generated households (an operation requested by the authors and performed by the Census and Statistics Department in Hong Kong). Of the households visited, only 1002 families had eligible adolescents or teenagers aged 9–19 living in the house of which 718 successfully completed the questionnaires, which corresponded to a 72% response rate. In January to March of 2010, the survey was re-administered to the respondents from Time 1. However, only 417 participated, yielding a 58% response rate at Time 2.

At Time 1, the sample consisted of 44% male respondents. The mean age of the whole sample was 14.46. The age distribution resembled the Hong Kong 2008 adolescent population census very closely. Of the 718 respondents, over 88% were high-school students or high-school graduates. In terms of family income, the mean was at the income bracket of US$1928–2571 per month. At Time 2, the panel sample contained 42% male respondents. The mean age of the panel sample was 15.28. Attrition analysis was also conducted and found that the dropouts at Time 2 were similar in social media use as stayers who completed the surveys at both times.

### 3.2. Measures

#### 3.2.1. Gratifications

Initially, items used in previous studies on the gratifications of the Internet such as sociability, instrumentality, reassurance, entertainment, acquisition, and time management were included in the survey questionnaire (Papacharissi & Rubin, 2000). Additional gratification items were included which related to the use of short message services, such as mobility, immediacy, affection, relaxation, and fashion or status (Leung, 2007). In this study, it is assumed that these motives would also apply to social media use. However, social media may involve other motives in addition to those gratification dimensions defined previously. Thus, a focus group was conducted to gather and refine gratification items directly related to social media use among a group of 27 children and adolescents. A total of 31 statements that reflected the different categories of reasons for using social media, especially for IM, blogs, Facebook, and online games, were tested. A 5-point Likert scale was used, with “1” meaning “strongly disagree” and “5” meaning “strongly agree” to rate each of the reasons presented for using the four social media listed.

A principal components factor analysis with a varimax rotation was run to determine the potential groupings of 31 items. Eight items with extremely low communalities and/or items that failed to load on any factors were removed. The analysis yielded six factors with an eigenvalue greater than 1.0, explained 63.43% of the variance, and the reliability alpha ranged from 0.61 to 0.85. The factors included “status-gaining”, which reflected how adolescents feel that social media use makes them feel important, cool, and fashionable, and how it increases their status and impresses people; “entertainment” revealed that social media use can be fun, entertaining, tension reducing, trendy, and used to pass time; “opinion expression” illustrated how social media is used to freely express opinions, participate in discussion, and to give input; “identity experimentation” indicated that the use of social media can help participants to escape from who they are, live out a fantasy, experience things they cannot in the real world, and to try out a new identity, especially in online games; “information-seeking” reflected how adolescents use social media to stay abreast of the news, gain immediate knowledge of major events, and to find out things they need to know about daily life; and “passing time” illustrated how adolescents use social media to avoid doing what they are supposed to be doing and to forget about their problems.

#### 3.2.2. Internet addiction

The 20-item Internet Addiction Scale (IAS) developed by Young (1998a) and additional items by Bianchi and Phillips (2005) were adapted to measure the level of Internet addiction in this study. A 5-point Likert scale was used with 1 = strongly disagree and 5 = strongly agree. Eighteen items survived the pre-test with two items that were low in communality, repetitive, or ambiguously worded being eliminated. The reliability for the scale as indicated by Cronbach's alpha was remarkably high at 0.90. The IAS was developed to collect responses from adolescents to identify Internet addiction symptoms and, as a composite index, to assess their overall level of Internet addiction. The mean score for the 18-item index was 2.58, and SD equaled 0.56.

To explore the Internet addiction symptoms, the principal components factor analysis yielded a five-factor structure and accounted for 64.62% of the total variance. The first factor was “preference for online” (*α* = 0.78) reflecting that addicts are more comfortable with computers than with people, more confident socializing online than offline, and feel better treated and safer relating to others in online relationships rather than face-to-face. “Loss of control” was the second factor (*α* = 0.82). It included four items characterizing that adolescents wonder what is happening online when they are not on, they feel they are missing out and lost if they cannot go online, and they are preoccupied with the Internet if they cannot connect for some time. “Preoccupation” was the third factor (*α* = 0.74). It consisted of three items illustrating that adolescents spend a good deal of time online, go online for longer than intended, and lose track of time online. The fourth factor was “withdrawal” (*α* = 0.81), reflecting that addicts use the Internet to talk with others when they feel isolated, lonely, and down. The fifth factor, “negative life consequences” (*α* = .65) contained four items indicating that adolescents find that excessive use of the Internet has caused trouble such as missing class, work and social events, and also to feelings of worthlessness when offline.

As a whole, this study identified five Internet addiction symptoms that were conceptually consistent with the theoretical origins described in the diagnostic criteria of pathological gambling in the *DSM-IV*. The original *DSM* measure for pathological gambling was based on 8 items; however, this study employed 18.

#### 3.2.3. Internet risks

This study adopted three risks generally encountered online by adolescents and were often researched in the literature (Leung & Lee, 2012a; Livingstone, 2008b; Livingstone & Helsper, 2010). The “internet harassment” dimension of the construct for Internet risks was measured with five items using a 5-point Likert scale with 1 = once a year, 2 = a few times a year, 3 = once or twice a month, 4 = once or twice a week, and 5 = almost every day. Respondents were asked if they had experienced harassment online. Sample items included the following: “received rude or nasty comments from someone while online”, “received obscene or indecent photo or picture”, and “received threatening or aggressive comments while online”. Cronbach's alpha equaled 0.74. “Privacy risk” was the second dimension measuring the Internet risks construct. Respondents were asked the following: in the past year, has a stranger: (1) Asked you to provide personal information while online? (2) Asked you to provide your personal photo while online? (3) Peddled merchandise or goods to you? and (4) Said he or she knew you but in fact you did not know him or her. Cronbach's alpha was 0.60 for this scale. Internet risk was also measured in the exposure to “pornographic or violent content” dimension using four items. Respondents were asked if they had visited or used websites intentionally or unintentionally such as pornographic sites, adult forums, sites with violent or sanguinary contents, and auction or online shopping sites. Cronbach's alpha was 0.60 for this scale. The scale used for both “privacy risk” and “pornographic or violent content” was 1 = yes and 0 = no.

#### 3.2.4. Social media use

To measure social media use, respondents were asked how often they used IM, blogs, Facebook, and online games each using a 5-point scale with 1 = never and 5 = very often. The reliability alpha was 0.72.

#### 3.2.5. Demographics

Social demographic variables were included in the present study as control variables: gender, age, and education.

## 4. Results

### 4.1. Surges and declines

Table 1 illustrates the trends of our measures among panel respondents. *T*-test results show the surge in social media use was particularly pronounced for Facebook (which indicates more than a full point rise on a 5-point Likert scale saying that adolescents' Facebook use has drastically increased over the period of one year) followed by a relatively small but significant surge in the use of IM. For the Internet addiction symptom questions, the significant increase was relatively modest, specifically in symptoms such as preoccupation and loss of control. As for gratifications-sought in social media use, a small but significant surge was also detected for expressing opinions and information-seeking. Among the three Internet risk measures, only privacy exposed indicated a significant, though small, upsurge in the one year period. In terms of decline, online gaming was the only item exhibiting a significant decline trend. We use these surges or declines as an opportunity to examine the effects of social media use, together with changes in gratifications, and Internet addiction symptoms, on Internet risks experienced by adolescents during a unique period in the phenomenal rise in popularity of social media in the Web 2.0 era.

**Table 1.** Summary of descriptive statistics on all key variables for panel in 2009–2010.

	2009 Time 1				2010 Time 2		*t*^a^
	Full sample(*N* = 718)		Panel (*N* = 417)		Panel (*N* = 417)		
	*M*/%	SD	*M*/%	SD	*M*/%	SD	
*Demographics*							
Gender							
Male	44.4%		41.1%		41.2%		–
Female	55.6%		58.9%		58.8%		–
Age	14.46	2.59	13.94	2.50	15.08	2.49	–
Education^b^	3.45	0.92	3.30	0.90	3.69	1.06	–
*Internet risks*							
Target of harassment^c^	1.58	0.65	1.51	0.06	1.49	0.55	−0.54
Privacy exposed^d^	1.23	1.27	1.12	1.18	1.35	1.27	3.13**
Pornographic/violentcontent consumed^e^	0.59	0.92	0.52	0.84	0.54	0.84	0.41
*Gratifications-sought*^f^							
Status-gaining	2.68	0.75	2.65	0.77	2.65	0.68	−0.23
Entertainment	3.48	0.68	3.49	0.70	3.43	0.66	−1.78
Expressing opinions	3.24	0.74	3.20	0.74	3.28	0.69	2.25*
Identity experimentation	2.87	0.78	2.82	0.79	2.78	0.77	−0.79
Information-seeking	3.85	0.59	3.83	0.60	3.96	0.54	3.96***
Passing time	2.85	0.90	2.81	0.91	2.79	0.87	−0.44
*Internet addiction symptoms*^f^							
Preference for online	2.73	0.83	2.73	0.82	2.69	0.81	−1.23
Loss of control	2.46	0.83	2.43	0.84	2.53	0.81	2.53*
Preoccupation	3.06	0.92	3.07	0.92	3.25	0.88	4.00***
Withdrawal	2.82	0.83	2.80	0.87	2.80	0.82	−0.04
Negative life consequence	1.90	0.65	1.87	0.61	1.85	0.54	−0.27
*Social media use*^g^							
IM	3.70	1.39	3.56	1.48	3.69	1.33	1.97*
Blogs	2.74	1.42	2.74	1.40	2.49	1.28	−0.92
Facebook	2.53	1.40	2.29	1.33	3.46	1.38	15.00***
Online games	2.88	1.34	2.93	1.40	2.79	1.31	−2.12*

^a^This was a *t*-test comparing the means in the panel sample between Time 1 and Time 2.

^b^Education was measured with 1 = Primary 1–3, 2 = Primary 4–6, 3 = Secondary 1–3, 4 = Secondary 4–5, 5 = Secondary 6–7, 6 = Associate degree, and 7 = bachelor degree.

^c^Scale used: 1 = less than once a year and 5 = almost every day on five items, and then taking the mean.

^d^Scale used: 1 = yes and 0 = no on three items, and then taking the mean.

^e^Scale used: 1 = yes, 0 = no on three items, and then taking the mean.

^f^Scale used: 1 = strongly disagree and 5 = strongly agree, and then taking the mean for each dimension.

^g^Scale used: 1 = never and 5 = very often on each item.

***p < .001.

**p < .01.

*p < .05

### 4.2. Predicting Internet addiction and symptoms

Our next analysis examined what influenced Internet addiction in general and Internet addiction symptoms in particular with data one year apart. Our model included the first-wave measures of social media use. However, it also included the demographic measures and measures of one other key predictor – gratifications-sought from social media use – that could have shaped each dependent variable. Table 2 presents the hierarchical regression results gained from regressing Internet addiction overall and the five Internet addiction symptoms at Time 2 on Internet addiction and addiction symptoms criterion variables, together with demographics, gratifications-sought, and social media use variables at Time 1.

**Table 2.** Summary of multiple hierarchical regression analyses regressing Time 2 Internet addiction symptoms on Time 1 Internet addiction symptoms criterion variables, demographics, gratifications, and social media use.

Predictors at Time 1	Internet addiction symptoms at Time 2											
	Internet addiction (composite)		Preference for online		Loss of control		Preoccupation		Withdrawal		Negative life consequence	
	*β*	Δ*R*^2^	*β*	Δ*R*^2^	*β*	Δ*R*^2^	*β*	Δ*R*^2^	*β*	Δ*R*^2^	*β*	Δ*R*^2^
Step 1:		0.26***		0.28***		0.22***		0.23***		0.22***		0.16***
T1 scores of criterion	0.51***		0.53***		0.47***		0.48***		0.47***		0.36***	
Gender (male = 1)	−0.09		0.01		−0.07		−0.05		−0.04		0.11*	
Age	0.04		−0.01		0.06		0.09		0.02		−0.06	
Education	0.07		−0.03		0.08		0.08		0.02		−0.06	
Step 2: T1 gratifications		0.04**		0.01***		0.07***		0.02***		0.03***		0.07***
Status-gaining	0.03		0.11*		−0.04		0.02		0.17***		0.15**	
Entertainment	0.22***		−0.01		0.17**		0.17***		−0.03		0.06	
Expressing opinions	0.04		−0.01		−0.00		0.01		0.04		0.03	
Identity experimentation	0.05		0.07		−0.03		0.10		0.01		0.12*	
Information-seeking	0.07		−0.04		0.10*		0.00		−0.01		−0.04	
Passing time	0.09		0.04		0.12*		0.04		0.03		0.12*	
Step 3: T1 social media use		0.02***		0.01***		0.02***		0.02***		0.00		0.00
IM	0.13***		0.04		0.13**		0.14**		0.07		0.07	
Blogs	0.03		0.02		−0.02		0.05		−0.03		−0.03	
Facebook use	−0.00		0.04		0.01		0.04		0.06		−0.04	
Online games	0.01		0.09*		−0.03		−0.02		−0.02		0.08	
*R*^2^		0.32		0.30		0.31		0.27		0.25		0.23
Adjusted *R*^2^		0.31		0.29		0.30		0.26		0.24		0.22
*F*		42.21***		50.25***		32.98***		46.25***		59.96***		22.36***

Note: *N* = 417.

***p < .001.

**p < .01.

*p < .05.

According to Finkel (1995), static score (or conditional change) models of this sort are generally superior as models of change to simple “unconditional” models of change scores.^1^ The coefficients we report here can also be interpreted as the causal effects of the independent variables on change in Internet addiction and addiction symptoms at Time 2, controlling for the respondents' initial level of Internet addiction and addiction symptoms at Time 1 (Finkel, 1995, p. 7).

As shown in Table 2, controlling for prior levels of Internet addiction and Internet addiction symptoms, demographics, and gratifications-sought at Time 1, we found significant effects of IM use on changes in overall Internet addiction as well as specific Internet addiction symptoms, in particular, loss of control and preoccupation, at Time 2. Similarly, controlling for prior level of preference for online Internet addiction symptoms, demographics, and gratifications-sought at Time 1, significant effect of online games on changes in preference for online Internet addiction symptoms at Time 2 was also found. Put another way, we can say that respondents who regularly use IM and/or play online games were more likely to be addicted to the Internet and exhibited more Internet addiction symptoms specifically in preference for online social interaction, loss of control, and preoccupation, controlling for initial levels of Internet addiction and addiction symptoms.

On the other hand, the extent of change in Internet addiction and addiction symptoms at Time 2 did vary across the initial levels of gratifications-sought in social media use, controlling for prior levels of Internet addiction and addiction symptoms at Time 1. In particular, the positive and significant effects of entertainment gratification on overall Internet addiction (*β* = 0.22, *p* < .001) at Time 2 indicate that, the more respondents sought entertainment gratification in their social media use at Time 1, the more likely they would be to report Internet addiction and exhibit symptoms at Time 2. Not surprisingly, the role of entertainment gratification was particularly pronounced in the case of exhibiting loss of control (*β* = 0.17, *p* < .01) and preoccupation (*β* = 0.17, *p* < .001) symptoms. By the same logic, controlling for prior level of preference for online and withdrawal at Time 1, the positive and significant effects of status-gaining gratifications on preference for online relationships (*β* = 0.11, *p* < .05) and withdrawal (*β* = 0.17, *p* < .001) indicate that adolescents who often seek status-gaining in the use of social media will be more likely to prefer online relationships and withdraw from interaction offline but going online when they feel isolated at Time 2. Similarly, controlling for prior levels of negative life consequences addiction symptoms at Time 1, the significant and positive effects of status-gaining (*β* = 0.15, *p* < .01), identity experimentation (*β* = 0.12, *p* < .05), and passing time (*β* = 0.12, *p* < .05) gratifications on negative life consequences indicate that adolescents whose use of social media was primarily for gaining status, experimenting with identity, and passing time will be more likely to suffer from negative life consequences at Time 2. Likewise, controlling for prior levels of loss of control addiction symptoms at Time 1, the significant and positive effects of entertainment (*β* = 0.17, *p* < .01), information-seeking (*β* = 0.10, *p* < .05), and passing time (*β* = 0.12, *p* < .05) gratifications on loss of control at Time 2 indicate that adolescents whose use of social media was primarily for entertainment, information-seeking, and passing time will be more likely to exhibit loss of control addiction symptoms. Variance ranged from 23% to 32%.

### 4.3. Predicting Internet risks

To examine what influenced individual level changes in Internet risks, we estimated another hierarchical regression model. This one predicted Internet risks at Time 2 as a function of Internet risks at Time 1, together with gratifications-sought, Internet addiction symptoms, and social media use also at Time 1. Three parallel regression analyses were conducted with being a target of harassment, privacy being exposed, and pornographic or violent content consumed used as dependent variables. The results in Table 3 indicate that, controlling for initial levels of being targeted for harassment at Time 1, the more adolescents sought status-gaining (*β* = 0.15, *p* < .01) and expressing opinion (*β* = 0.11, *p* < .05) gratifications in social media through blog use (*β* = 0.12, *p* < .01) at Time 1, the more likely they would be to experience an increase in being a target of harassment over this one year period. None of the other variables in the model were significant predictors of changes in being a target of harassment, controlling for other factors. Similarly, controlling for initial levels of privacy exposure, the more adolescents sought expressing opinions (*β* = 0.13, *p* < .01) gratification through Facebook use (*β* = 0.16, *p* < .01) resulting in experiencing more Internet addiction symptoms such as withdrawal (*β* = 0.11, *p* < .05) and negative life consequences (*β* = 0.13, *p* < .01), the more privacy risks they would be exposed to at Time 2. Interestingly, controlling for initial levels of pornographic or violent content consumed, results show that being male (*β* = 0.30, *p* < .001), upper class (*β* = 0.11, *p* < .01), and gratified mostly in identity experimentation (*β* = 0.11, *p* < .01) in their use of social media at Time 1, the more likely those adolescents would be to take risks in their consumption of pornographic and/or violent content online at Time 2. Again, no other variables were significant predictors of change after controlling for other factors. Total *R* ^2^ ranged from 19% to 49%.

**Table 3.** Summary of multiple hierarchical regression analyses regressing Time 2 Internet risks on Time 1 Internet risks criterion variables, demographics, gratifications, Internet addiction symptoms, and social media use.

Predictors at Time 1	Internet risks at Time 2					
	Target of harassment		Privacy exposed		Pornographic/violentcontent consumed	
	*β*	Δ*R*^2^	*β*	Δ*R*^2^	*β*	Δ*R*^2^
Step 1:		0.13***		0.18***		0.47***
T1 scores of criterion	0.36***		0.42***		0.53***	
Gender (male = 1)	0.07		−0.09		0.30***	
Age	0.04		0.01		0.05	
Education	0.05		−0.01		0.11**	
Step 2: T1 gratifications		0.05***		0.02***		0.02***
Status-gaining	0.15**		0.07		0.04	
Entertainment	0.04		0.04		−0.02	
Expressing opinions	0.11*		0.13**		0.05	
Identity experimentation	−0.06		0.03		0.11**	
Information-seeking	−0.01		0.02		0.01	
Passing time	−0.06		0.01		0.03	
Step 3: T1 Internet addictionsymptoms		0.00		0.01***		0.00
Preference for online	0.01		0.01		−0.00	
Loss of control	0.04		0.01		0.04	
Preoccupation	−0.00		0.06		0.04	
Withdrawal	0.07		0.11*		0.01	
Negative life consequence	−0.01		0.13**		0.01	
Step 4: T1 social media use		0.01***		0.02***		0.00
IM	0.06		0.01		0.04	
Blogs	0.12**		0.02		−0.01	
Facebook use	0.07		0.16**		0.02	
Online games	0.01		−0.06		−0.06	
*R*^2^		0.19		0.23		0.49
Adjusted *R*^2^		0.18		0.22		0.48
*F*		20.85***		20.88***		83.63***

Note: *N* = 417.

***p < .001.

**p < .01.

*p < .05.

## 5. Discussions

### 5.1. Effects of gratifications-sought on Internet addiction, symptoms and Internet risks

The results presented in Table 2 shed light on some of the factors that influenced Internet addiction and addiction symptoms over the course of the following year. To begin with, the results showed that the more adolescents find social media use gratifying at Time 1, the more likely than others they will be to report Internet addiction and symptoms at Time 2. The magnitude of the effect from gratifications-sought varied in understandable ways. Its impact was greatest from entertainment gratifications, followed by status-gaining. The weakest impact was from information-seeking followed by identity experimentation, and passing time. Such results indicate that adolescents who scored highly in entertainment and status-gaining gratifications at Time 1 might be most likely to develop pathological Internet use and exhibit addiction symptoms one year later.

Specifically, it is interesting to note that adolescents who habitually rely on social media to satisfy their entertainment, passing time, and information-seeking needs are often those who have difficulty controlling their Internet time. Similarly, those who routinely rely on social media to impress people, gain status, try out a new identity, avoid responsibilities, and to forget their problems are those who find themselves more vulnerable to suffering academically and socially (Vrocharidou & Efthymiou, 2012). Such behavioral patterns are good warning signs for parents and teachers to forecast the type of addiction symptoms adolescents will develop.

With respect to the relationship between gratifications-sought and Internet risks, one might think that increased Internet risks (becoming a target of harassment and having privacy exposed) is the *obvious* cost in the process of seeking gratification especially entertainment, information-seeking, and passing time from social media. However, we did not find any evidence that these motivations explained individual level changes in Internet risks. Those who were motivated by these social psychological needs were no more or less likely to exhibit increasing Internet risks than those who did not. However, our results suggest that some of the increased Internet risks may be due to a series of *proactive*, *self-instigated*, and *purposive* social media use to meet a higher order of social psychological needs. This higher order of needs include status-gaining or recognition, expressing opinions or engaging in intellectual dialogue as well as identity experimentation, or trying out new identities to escape from reality. Adolescents who seek these higher-order instrumental gratifications at Time 1, as compared with the *passive* or *habitual* gratifications such as entertainment or fun seeking, escapism, passing time, or browsing websites (Leung, 2007; Rubin, 1984), may be more likely to become targets of harassment for cyberbullies and to inadvertently exposing private information without realizing the possible risks (Keith & Martin, 2005).

### 5.2. Effects of social media use on Internet addiction, symptoms, and Internet risks

One finding that stands out is the consistent effect of IM social media use on overall Internet addiction and specifically on loss of control and preoccupation addiction symptoms. For each of the dependent variables, this effect was positive and statistically significant at the 0.05 level or better. Such results do indicate that, all else being equal, IM use at Time 1 was positively associated with Internet addiction and some of the symptoms at the peak of the surge at Time 2. Similarly, online gaming also significantly predicted addiction symptoms in preference for online socializing albeit to a much lesser degree.

However, we found no evidence of a relationship between Facebook or blog use and either Internet addiction overall or any particular Internet addiction symptom exhibited at Time 2. The coefficient for Facebook and blog use did not attain statistical significance for any of the dependent variables. Such results suggest that IM and online gaming, despite IM having a significant surge and online gaming a significant decline in popularity over the one year period (Table 1), seem to have a stronger effect on addiction than blog and Facebook use for adolescents. Over the study period, adolescents may be susceptible to developing the symptoms of losing control and being preoccupied online when they have been heavy users of IM and online games. Others may develop a preference for the online world as they may feel safer, more comfortable, better treated, and more confident socializing online (Caplan, 2003; Leung, 2011; Thayer & Ray, 2006). One possible explanation is that online games such as massively multiplayer online role-playing games can be very addictive as adolescents can role-play a character, immerse themselves in a fantasy world, and become well-known in online games (Frostling-Henningsson, 2009; Smahel, Blinka, & Ledabyl, 2008). Despite the actual decline in online gaming over the study period, the effect remains significant after controlling for the initial level of preference for online social interaction, demographics, and gratifications at Time 1. Similarly, the effects from IM use on loss of control and preoccupation symptoms can be explained by the increasing popularity of IM over the one year period. IM's trendiness and its role as the reciprocal norm to communicate with peers have afforded adolescents to develop these two symptoms and ultimately Internet addiction. It is also interesting to note that affordances such as mobility and ubiquity that adolescents enjoy through their use of gadgets such as smart phones, iPad, and tablets to access social media platforms may have also accelerated the addiction rate.

In analyzing the predictors for Internet risks, controlling for the initial level of Internet risks at Time 1, it is noteworthy that the previous pattern of blog use significantly predicted the likelihood of being targeted for harassment. Similarly, the pattern of Facebook use predicted the chances for participants having had their privacy exposed one year later. Such findings can be explained by the fact that blogs and Facebook are platforms for freely expressing opinions. However, such freedom may also increase the probability of becoming targets for cyberbullies to engage in personal attacks, harassment, and even unwelcome sexual solicitation. Also, Facebook is a place where most adolescents misperceive as safe and they even boast about having a large amount of personal information being exhibited for the whole world to see without being cautious about disclosing too much. Therefore, one important contribution of the study is the notion that heavy social media use, especially in Facebook and blogs, may be an early warning sign to differentiate those who should be monitored more closely to keep them from falling victim to unnecessary Internet risks.

### 5.3. Effects of Internet addiction and symptoms on Internet risks

The initial usage pattern of social media and gratifications-sought had significant, albeit small, effects on Internet risks over the one year period. It is also interesting to note that Internet addiction symptoms (especially withdrawal and negative life consequences) influence the amount of private information exposed. This is understandable as adolescents who suffer from worsening academic performance and deteriorating offline social relationships due to Internet addiction may spend much more time online on Facebook when they feel isolated or depressed. The more time they spend online, the more likely they are to self-disclose and be solicited for private information by strangers.

## 6. Implications

The current study extends the literature by examining the complex relationships between being a victim of Internet risks and gratifications-sought, addiction symptomatology, and social media use. The major contribution of this study is that, with their explanatory power, these predictors could be used to identify those adolescents who are most vulnerable and likely to become addicted. They may also identify those most likely to develop Internet addiction symptoms and become most susceptible to experiencing Internet risks based on their current or previous gratifications-sought and their addiction symptoms exhibited in Internet use. Due to the longitudinal nature of this study, it is reasonable to conclude that social media use (i.e. IM and online games) actually influences the development of Internet addiction. Similarly, blog and Facebook use also have an effect on Internet risks especially becoming a target for harassment and inadvertently exposing privacy. Overall, this research suggests that specific and targeted efforts may be needed to counter Internet risks in order for children and adolescents to benefit from the many opportunities offered by the Internet, especially from social media.

The result that reliance on IM and online games, but not Facebook or blogs, was significantly related to Internet addiction poses an interesting question. Future research into adolescents could usefully consider why one form of social media is more addictive than another. With newer social media introduced into the market such as Twitters, WhatApp, WeChat (being very popular in China), and YouTube, future research, preferably cross-cultural, should examine more closely the differential gratifications-sought and addiction symptoms arising from these popular social media platforms. This may provide different early warning signs for parents and educators to reduce adolescents' probability of exposure to Internet risks. Being a target for harassment, having privacy exposed, and consumption of pornographic or violent content are pertinent Internet risks that adolescents encounter every day (Finkelhor, Mitchell, & Wolak, 2000). Future research should investigate other risk types such as cyber-bullying, identity theft, cyber-aggression, cyber-stalking, and sex solicitation.

## 7. Limitations and suggestions for future research

First, this study infers social media effects from survey findings, rather than capturing them through experimental control. Our analyses control for the most likely sources of spurious relationships between social media use and addiction symptoms and Internet risks. Nonetheless, we cannot be certain of the direction of these effects. In addition, we cannot link our respondents to the specific content, activities, and goals when they engaged in social media; thus, one major limitation of the study is that we can only speculate about the reasons behind the differences in the effect of social media over time and across different platforms. We do, however, see our account as plausible and consistent with the available evidence. Future studies should address specific functions, goals, and content they engaged in on different social media for an accurate account of the effects of the activities on Internet addiction and Internet risks.

## Note

1. The unconditional change score model assumes that prior values of the dependent variable have no influence on current values or change in the dependent variable, a potentially problematic assumption in the case at hand.
